# Optimization of Enzymatic Saccharification of Alkali Pretreated *Parthenium* sp. Using Response Surface Methodology

**DOI:** 10.1155/2014/764898

**Published:** 2014-05-12

**Authors:** K. Pandiyan, Rameshwar Tiwari, Surender Singh, Pawan K. S. Nain, Sarika Rana, Anju Arora, Shashi B. Singh, Lata Nain

**Affiliations:** ^1^Division of Microbiology, Indian Agricultural Research Institute, New Delhi 110012, India; ^2^School of Civil & Mechanical Engineering, Galgotias University, Greater Noida, Uttar Pradesh 201306, India; ^3^Division of Agricultural Chemicals, Indian Agricultural Research Institute, New Delhi 110012, India

## Abstract

*Parthenium* sp. is a noxious weed which threatens the environment and biodiversity due to its rapid invasion. This lignocellulosic weed was investigated for its potential in biofuel production by subjecting it to mild alkali pretreatment followed by enzymatic saccharification which resulted in significant amount of fermentable sugar yield (76.6%). Optimization of enzymatic hydrolysis variables such as temperature, pH, enzyme, and substrate loading was carried out using central composite design (CCD) in response to surface methodology (RSM) to achieve the maximum saccharification yield. Data obtained from RSM was validated using ANOVA. After the optimization process, a model was proposed with predicted value of 80.08% saccharification yield under optimum conditions which was confirmed by the experimental value of 85.80%. This illustrated a good agreement between predicted and experimental response (saccharification yield). The saccharification yield was enhanced by enzyme loading and reduced by temperature and substrate loading. This study reveals that under optimized condition, sugar yield was significantly increased which was higher than earlier reports and promises the use of *Parthenium* sp. biomass as a feedstock for bioethanol production.

## 1. Introduction

In the last few decades, the demand for alternative fuel sources is accelerated due to the excessive consumption of fossil fuels [1]. Currently, ethanol production process uses crops such as sugar cane and corn but they have social issues associated with the exploitation of potential food or feed resources [2]. Therefore, the utilization of nonfood biomass, that is, lignocellulosic biomass, is creating interest worldwide. The lignocellulosic biomass has the advantage of huge availability, being economical, and reduced emissions of greenhouse gases and does not have the socioeconomic concerns regarding the use of food resources. These factors make them one of the most promising technological approaches available for supplementing the current source of transportation fuel. Effective conversion of recalcitrant lignocellulosic biomass to ethanol includes five subsequential steps: (1) biomass pretreatment, (2) cellulose hydrolysis (saccharification), (3) fermentation of hexoses, (4) separation, and (5) effluent treatment [3].

*Parthenium* sp., belonging to the family Asteraceae, is native to the American tropics and commonly known as carrot weed or gajar ghas. At present this invasive weed has infested about 35 million ha of land in India since its first introduction in 1955 [4]. It is able to grow on wide range of soil types ranging from sandy to heavy clay soil, but better growth is observed in moist type of soil. It occurs in areas with summer rainfall greater than 500 mm per annum [5]. The excessive growth rate and wider adaptability of this weed without any fertilizer input shows it as a potential renewable source of lignocellulosic biomass available for ethanol production.

Pretreatment process is essential for removal of lignin and hemicelluloses to reduce cellulose crystallinity and increase the porosity of biomass [6]. Enzymatic saccharification of cellulosic biomass has been considered as an environmentally friendly process that replaces harsh acid treatment for saccharification [7]. The main bottleneck for the commercialization of bioethanol is due to high costs of the two processes, pretreatment and enzymatic hydrolysis [8].

Saccharification is an important step for maximum sugar yield, with enzyme, substrate loading, pH, and temperature constituting important parameters for optimization of saccharification process. Optimization of saccharification process is highly challenging as it is necessary to obtain high yield of monomeric sugars which can be converted into bioethanol by fermentation process. Optimization of multifactorial system by conventional techniques is generally done with one-factor at a time. However, this type of method is time consuming and does not reveal the interactive effects between the variables [7]. RSM is a statistical technique for the modeling and optimization of multiple variables, which determines the optimum process conditions through combining experimental designs with interpolation by first- or second-order polynomial equations in a sequential testing procedure [9].

In the present study,* Parthenium* biomass was used as a source of fermentable sugars by subjecting it into mild alkali pretreatment. An attempt was also made to identify the optimum process conditions for maximum sugar release from* Parthenium *biomass by using central composite rotatable design (CCRD) and analyzing the influence and interactions of variables during saccharification.

## 2. Materials and Methods

### 2.1. Pretreatment and Compositional Analysis of Parthenium

The* Parthenium *biomass was collected from Indian Agricultural Research Institute (IARI) farm in the month of May, air dried and chopped into small size (2–5 cm), and stored in an airtight polyethylene bag at room temperature until further use. The pretreatment was carried out using 1% NaOH as described previously [10] and washed with distilled water to bring down pH 7.0. The cellulose content of raw and pretreated biomass was determined by the method described by Updegraff [11]. Pentosans, klason lignin, moisture, and ash contents were determined according to TAPPI [12] method.

### 2.2. Saccharification of Pretreated Substrate

Saccharification of pretreated substrate was carried out as described by NREL [13]. Briefly, pretreated substrate was placed in 50 mL screw capped bottles on a rotary shaker. A set up comprising 10 mL reaction mixture in 50 mM sodium citrate buffer was prepared according to the experimental design, with supplementation of 100 *μ*L of sodium azide (2%), to prevent microbial contamination. The enzyme complex used for hydrolysis was Accellerase 1500 (52.0–62.0 FPU/mL). Samples were taken from the reaction mixture at different time intervals and centrifuged at 10000 rpm for 5 min. The supernatant was used for analysis of reducing sugar by HPLC as described previously [14] using Waters HPLC and Aminex HPX-87H column. Saccharification efficiency was calculated by the following formula as described by NREL [13]:

(1) Saccharification(%) =Reducing  sugars  released(mg)×0.9Carbohydrate  content  in  pretreated  biomass×100.

### 2.3. Design of Experiment

The sugar yield of enzymatic hydrolysis in terms of saccharification efficiency was taken as the response influenced by many potential variables. In this study, a central composite rotatable design (CCRD) was employed to determine the effect of independent variables on response and to optimize the enzymatic hydrolysis. Thirty runs of experiment were formed by Design Expert 8.0.7.1 version (Stat-Ease, Inc., Minneapolis, USA) with six replications at the central point, eight replications at the axial points, and sixteen replications at the factorial points. The variables include temperature (*A*), pH (*B*), enzyme loading (*C*), and substrate loading (*D*). The coded and decoded values are listed in Table 1.

Using Design Expert 8.0.7.1, all the statistical and mathematical analysis of the results was done to evaluate the effects of variables and their interactions. Three dimensional surface plots were drawn to show the effects of independent variables on response and a quadratic polynomial equation was proposed to describe the mathematical relationship between the variables and the response. The significance of the model was evaluated by determination of *R* ^2^ and adjusted *R* ^2^ coefficient. An experiment was also conducted to confirm the predicted optimum response using the selected optimum values of the four variables.

## 3. Results

### 3.1. Compositional Analysis of Parthenium

The compositional analysis of raw sample revealed that the biomass contains cellulose (308.03 ± 0.6 mg/g); pentosans (164.45 ± 0.2 mg/g), and klason lignin (181.28 ± 1.0 mg/g). The pretreatment of substrate with 1% NaOH increased the proportion of cellulose and pentosans by 30.5 and 22%, respectively, and reduced the lignin content by 16.6% in the biomass (Table 2).

### 3.2. Saccharification of Pretreated Substrates

Enzymatic hydrolysis of alkali pretreated* Parthenium* biomass under unoptimized conditions using commercial enzyme complex, Accellerase 1500 at 50°C and pH 4.8, resulted in maximum sugar release of 513 mg/g of dry substrate (76.6%) after 48 h of saccharification.

### 3.3. Optimization of Saccharification Parameters for Increased Sugar Yield

#### 3.3.1. Development of a Model for Enzymatic Saccharification

On the basis of initial results from enzymatic hydrolysis, the conditions for optimization of hydrolysis are as follows: temperature, 45–65°C; pH, 4-5; enzyme loading, 0.2–1.0 mL; substrate loading, 0.1–0.5 g, which are summarized in Table 3. The buffer used for enzymatic hydrolysis was sodium citrate buffer but the range of pH was modified according to the experimental design.

The coefficient of determination (*R* ^2^) of the model was 0.96 (Table 4) while coefficient of variation (CV%) and standard deviation (SD) were 12.91 and 5.53, respectively. The *S*/*N* ratio was found to be 18.62.

The model *F*-value of 26.51 implied that the model was significant and there was only a 0.01% chance that the model *F*-value could occur due to noise (Table 4). The “*P* value” for the model was <0.0001 while the *P* value for model terms *A*, *C*, *D*, *AD*, *A* ^2^, *B* ^2^, and *C* ^2^ were less than 0.05. *AD* (temperature to substrate loading) was also a significant variable with *P* value of 0.0289.

The overall second-order polynomial equation (2) describes the relationship between the variables and the sugar yield from enzymatic hydrolysis of pretreated* Parthenium* sp. in terms of coded values

(2) Y=+  54.73−17.84×A−1.33×B+6.94×C −4.71×D−0.83×AB−2.32×AC+3.34 ×AD+1.50×BC+0.21×BD−0.93×CD −3.78×A2−3.24×B2−0.94×C2−6.91×D2,

where the coded variables were *Y*—saccharification (%); *A*—temperature (°C); *B*—pH; *C*—enzyme loading (mL); *D*—substrate loading (g).

#### 3.3.2. Influence of Variables on Saccharification Yield

**Figure 1:**
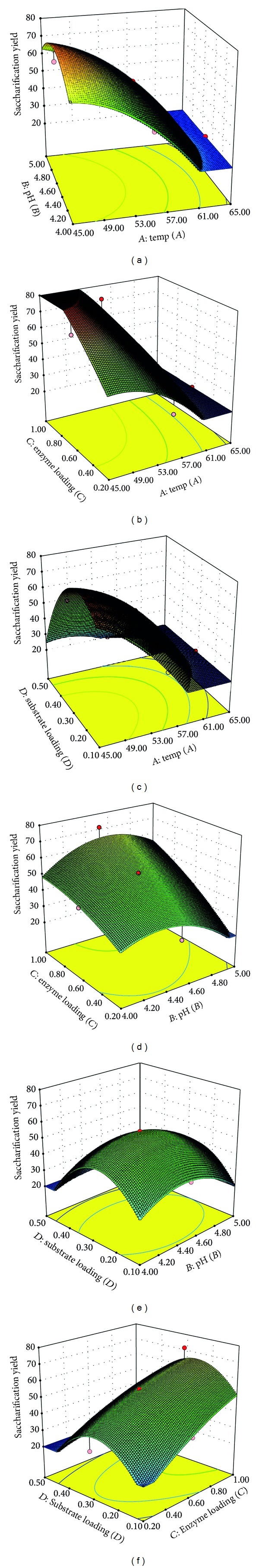
Response surface plots of central composite design for optimization of the enzymatic hydrolysis of alkali pretreated* Parthenium* sp. Figure shows the interaction between (a) temperature and pH; (b) temperature and enzyme loading; (c) temperature and substrate loading; (d) pH and enzyme loading; (e) pH and substrate loading; and (f) enzyme loading and substrate loading.

The effect of incubation temperature and pH, when enzyme loading and substrate loading were at their central level, 0.6 mL and 0.3 g, respectively, are shown in Figure 1. The increase in temperature resulted in low saccharification yield. An improvement in saccharification yield was observed with increase in enzyme loading at optimum pH and substrate loading (Figure 1(b)). The increase in substrate loading beyond 0.4 g resulted in reduction of saccharification yield (Figure 1(c)).

Figures 1(d)–1(f) show the interaction of variables: enzyme loading, substrate loading, and pH and thus interactions on enzymatic saccharification. The pH did not show any significant effect on response while interacting with enzyme loading and substrate loading. The influence was solely due to the interacting variable, that is, either enzyme or substrate loading.

#### 3.3.3. Optimization of Saccharification Yield (%)

On the basis of experimental design and developed model, the optimal conditions to maximize the saccharification yield were obtained. The predicted maximum saccharification yield was 80.08% during enzymatic hydrolysis under the optimum conditions, that is, temperature—50°C; pH—4.53; enzyme loading—0.80 mL (7 FPU/g); substrate loading—0.24 g (Table 5).

To validate the predicted saccharification yield, an experiment was conducted in triplicate with the above mentioned optimum conditions of each variable as developed by the model. The experimental result of response (saccharification %) for pretreated* Parthenium* sp. was 85.80% and it was in good agreement with predicated value of 80.08% for saccharification yield (Table 5). HPLC analysis revealed that the saccharified material contains mainly glucose, xylose, and arabinose (see Figure 1 in Supplementary Materials available online at http://dx.doi.org/10.1155/2014/764898).

## 4. Discussion

High growth rate without any economic input, vast availability, and high glucan content (60.2%) makes lignocellulosic weedy biomass like* Parthenium* an attractive source to supplement the feedstock supply for bioethanol production. Many weedy lignocellulosic biomasses like* Lantana, Eichhornia, Saccharum, *and* Prosopis *have been exploited as feedstock for biofuel purposes [20–23]. The major bottlenecks in commercial production of bioethanol are recalcitrant nature of raw material, high cost of enzymes for saccharification, and nonavailability of cofermenting (hexoses and pentoses) yeasts. Many efficient methods including physicochemical and biological methods have been used successfully for removal of lignin and thus increasing the saccharification efficiency of different lignocellulosic biomasses [10, 18]. Many efficient cellulases are available in market (Accellerase, Celluclast, and Novozyme 188) but the cost of these enzymes is still very high for an economically feasible process. The composition of different biomasses varies considerably and requires specific pretreatments and saccharification conditions for maximum production of fermentable sugars. Therefore, it is imperative to optimize the pretreatment conditions along with enzymatic hydrolysis variables in order to achieve maximum saccharification efficiency. Among various pretreatment methods tried for* Parthenium*, alkali (1% NaOH) treatment showed high recovery of acid perceptible polymerised lignin (7.53 ± 0.5 mg/g) and significantly higher amount (513.1 ± 41.0 mg/gds) of reducing sugars [10]. In this study, an attempt was made to optimize saccharification parameters such as temperature, pH, enzyme, and substrate loading by using RSM. Accellerase 1500 used in the study is one of the leading enzyme cocktail from DuPont-Genencor for cellulose hydrolysis [24].

RSM, a collection of statistical and mathematical techniques, is normally used for modeling and analyzing problems in which several variables influencing the response of interest may be tested and the aim is to optimize the response [9]. Saccharification efficiency, the response which is influenced by temperature, pH, enzyme loading, and substrate loading using central composite rotatable design (CCRD), was evaluated. CCRD has been the design of choice for optimization studies in biochemical processes due to its obvious advantages of rotability and the ability to analyse the interaction effects over mixture design [2].

Conventional optimization approach using one variable at a time (OVAT) is time consuming and also ignores the interaction of various variables used. The “*P* value” for the model used was <0.0001, which indicated that the model was statistically significant and the *P* value for model terms *A*, *C*, *D*, *AD*, *A* ^2^, *B* ^2^, and *C* ^2^ were less than 0.05 indicating that they were the significant variables influencing the response (saccharification %) than the others. The absence of interactions between variables (*P* > 0.05) except for *AD* can be assumed to be an additive effect of these variables on the response. *AD* was also a significant variable with *P* value of 0.0289 demonstrating that there was interaction existing between temperature and substrate loading.

The *R* ^2^ value (0.96) was in good agreement with the adjusted *R* ^2^ value (0.93) and well adapted to the response, also the predicted *R* ^2^ value (0.78) was in reasonable agreement with the adjusted *R* ^2^ value. From the above *R* ^2^ value, it was concluded that only 4% of the variation for response could not be explained by the model. The coefficient of variation (CV%) of 12.91 and standard deviation (SD) of 5.53 were relatively low and acceptable. The *S*/*N* ratio of 18.62 indicated the adequate signal and the model can be used to navigate the design space.

To analyze the interaction of variables and to determine the optimum value of each variable for maximum saccharification yield, three dimensional response surface curves were drawn against two experimental variables while the other variables were maintained constant at their central level. Among the variables studied for optimization, enzyme loading, substrate loading, and temperature have more effects on the saccharification yield of pretreated* Parthenium* sp.

The decreased saccharification efficiency on increasing temperature could partially be explained by the loss of enzyme activity due to thermal inactivation [25] while the change in pH showed a minimum effect on response. This might be due to the adaptability of cellulase enzyme complex for the pH range from 4 to 5 which was selected in this study.

Enzyme loading has been reported to be one of the most important factor and generally high enzyme loading results in better hydrolysis probably by increasing the rate and yield of enzymatic hydrolysis [25, 26]. However, increase in the enzyme loading of >0.8 mL have no significance in the saccharification yield which could be due to the decrease in extent of adsorbed enzyme, transformation of cellulose structure into a less digestible form, and inhibition of enzyme activity by accumulated hydrolysis products [27]. Improper mixing due to high substrate loading might have hindered enzymatic hydrolysis resulting in lower saccharification efficiency at higher substrate load [28].

Optimization of saccharification was carried out numerically by using Design Expert software, version 8.0.7.1, to evaluate the optimum values for each variable from the model. The experimental result of response (saccharification %) for pretreated* Parthenium* sp. was 85.80% and it was in good agreement with the predicated value of 80.08% for saccharification yield and showed that the model was useful for predicting the optimal conditions for variables influencing the saccharification yield as indicated by the good agreement between experimental (85.80%) and predicted values (80.8%). The reducing sugar yield and saccharification efficiency using alkali treated biomass under optimized conditions (574 mg/gds, 85.80%) was about 1.1-fold higher than unoptimized conditions (513 mg/gds, 76.7%) [10].

Several studies have been reported saccharification using various pretreatment methods for different lignocellulosic materials. The saccharification yield during enzymatic hydrolysis of different weedy lignocellulosic biomass as reported by other workers is summarized in Table 6. The results revealed the superiority of* Parthenium* biomass in yielding highest amount of sugars under optimized conditions. In addition to this, it also affirms probable reduction in cost of saccharification with minimum energy requirement since pretreatment was carried out at room temperature (40–45°C). It also confirms the validity of RSM as compared to conventional methods of optimization [7]. The optimum process parameters along with mild alkali pretreatment at room temperature have the additional advantage of producing a clean substrate which is highly digestible and rich in cellulose and pentosans [29]. In addition to this, availability of substrate without overhead costs makes the finding of this investigation a promising approach for bioethanol production.

## 5. Conclusion

The potential of* Parthenium* sp. as a source of fermentable sugar for bioethanol production was evaluated by estimating the sugar yield during enzymatic saccharification. To optimize the experimental variables of enzymatic hydrolysis for maximization of saccharification yield, CCD was employed under RSM. This experimental design converts the process variable correlations into a mathematical model which predicts the location of response. From the results it can be concluded that saccharification yield was mainly enhanced by enzyme loading in the given range and inversely affected by temperature and substrate loading. The pH had a neutral effect on the response. Under the optimum conditions, the predicted saccharification yield of 80.08% was in good agreement with the experimental results of 85.80% and validated the model generated by RSM.

## Supplementary Material

The optimized condition predicted by RSM was validated to evaluate saccharification yield (%) of pretreated *Parthenium* sp. The HPLC analysis of saccharified hydrolysate under optimized condition showing the presence of three major peaks corresponding to glucose, xylose and arabinose. The chromatogram also represents the major contribution of glucose in the hydrolysate followed by xylose and arabinose. The quantitative analysis showing the 85.80% of saccharification yield which is in good agreement with the predicted value (80.08%).

## Figures and Tables

**Table 1:** Coded and decoded values for each variables of central composite design (CCD).

Variables	Coded levels of the experimental variables				
	−*α*	−1	0	+1	+*α*
Temperature (°C)	45	50	55	60	65
pH	4.0	4.25	4.5	4.75	5.0
Enzyme loading (mL)	0.2	0.4	0.6	0.8	1.0
Substrate loading (g)	0.1	0.2	0.3	0.4	0.5

**Table 2:** Compositional analysis of raw and alkali pretreated *Parthenium* sp.

Composition	Raw sample (mg/g)	Alkali treated (mg/g)
Cellulose	308.03 ± 0.6	402.03 ± 0.4
Pentosans	164.45 ± 0.2	200.34 ± 0.3
Klason lignin	181.28 ± 1.0	151.92 ± 0.5
Ash	87.12 ± 0.3	61.16 ± 0.1

Data represent mean ± SD.

**Table 3:** Experimental design and results of CCD for enzymatic hydrolysis of pretreated *Parthenium* sp.

Run number	*A*: temperature (°C)	*B*: pH	*C*: enzyme loading (mL)	*D*: substrate loading (g)	Saccharification efficiency (%)	
					Experimental	Predicted
1	50	4.25	0.8	0.2	72.52	75.16
2	60	4.25	0.4	0.4	17.38	20.40
3	50	4.75	0.4	0.4	46.88	39.52
4	55	4.5	0.6	0.3	54.73	54.73
5	60	4.25	0.8	0.2	25.97	29.81
6	50	4.25	0.4	0.2	61.51	57.78
7	55	4.5	0.6	0.1	35.67	36.52
8	60	4.25	0.8	0.4	23.21	24.78
9	55	4.5	1.0	0.3	71.52	64.83
10	50	4.25	0.8	0.4	59.14	56.79
11	45	4.5	0.6	0.3	68.50	75.31
12	60	4.75	0.8	0.2	22.16	28.07
13	55	4.5	0.2	0.3	28.41	37.07
14	55	4.5	0.6	0.3	54.73	54.73
15	50	4.75	0.8	0.2	83.27	76.73
16	60	4.75	0.8	0.4	23.67	23.87
17	60	4.75	0.4	0.2	15.15	13.98
18	60	4.25	0.4	0.2	26.29	21.71
19	65	4.5	0.6	0.3	8.78	3.94
20	55	4.5	0.6	0.5	16.52	17.66
21	55	5.0	0.6	0.3	38.12	39.10
22	55	4.0	0.6	0.3	43.45	44.44
23	55	4.5	0.6	0.3	54.73	54.73
24	50	4.75	0.4	0.2	53.37	53.35
25	55	4.5	0.6	0.3	54.73	54.73
26	55	4.5	0.6	0.3	54.73	54.73
27	50	4.75	0.8	0.4	53.05	59.18
28	50	4.25	0.4	0.4	47.47	43.12
29	60	4.75	0.4	0.4	14.59	13.50
30	55	4.5	0.6	0.3	54.73	54.73

**Table 4:** ANOVA for quadratic response surface model (RSM) from enzymatic saccharification of pretreated *Parthenium* sp.

Source	Sum of squares	Degree of freedom	Mean square	*F* value	*P* value (Prob > *F*)
Model	11340.56	14	810.04	26.51	<0.0001
*A*: temperature	7640.87	1	7640.87	250.04	<0.0001
*B*: pH	42.69	1	42.69	1.40	0.2556
*C*: enzyme loading	1155.07	1	1155.07	37.83	<0.0001
*D*: substrate loading	533.46	1	533.46	17.45	0.0008
*AB*	10.91	1	10.91	0.36	0.5591
*AC*	86.26	1	86.26	2.82	0.1136
*AD*	178.29	1	178.29	5.83	0.0289
*BC*	35.91	1	35.91	1.18	0.2955
*BD*	0.69	1	0.69	0.023	0.8823
*CD*	13.78	1	13.78	0.45	0.5121
*A* ^2^	391.03	1	391.03	12.80	0.0028
*B* ^2^	287.84	1	287.84	9.42	0.0078
*C* ^2^	24.47	1	24.47	0.80	0.3850
*D* ^2^	1310.41	1	1310.41	42.88	<0.0001
Residual	458.38	15	30.56		
Lack of fit	458.38	10	45.84		
Pure error	0.00	5	0.00		
Total	**11798.95**	**29**			

SD	5.53		*R* ^2^	0.96	
Mean	42.83		Adjusted *R* ^2^	0.93	
CV(%)	12.91		Predicted *R* ^2^	0.78	

**Table 5:** Optimum values of variables for enzymatic saccharification of pretreated *Parthenium* sp.

Variables	Goal	Optimum levels	Desirability
Temperature (°C)	In range	50	0.957
pH	In range	4.53	-do-
Enzyme loading (mL)	In range	0.80	-do-
Substrate loading (g)	In range	0.24	-do-
Response	Goal	Predicted value	Observed value

**Saccharification ** **(%)**	**Maximize**	**80.08**	**85.80**

**Table 6:** Comparison of sugar release from different weedy lignocellulosic biomass after enzymatic saccharification.

S. no.	Biomass	Pretreatment	Sugar release (mg/gds)	Enzyme used	Reference
1	Giant reed	Dilute acid	481.6	Celluclast 1.5 L and Novozyme-188	[15]
2	Switch grass	Dilute acid	440.00	Commercial cellulase	[16]
3	*Parthenium *	Alkali	513.1	Accellerase 1500	[10]
4	*Parthenium *	Biological	485.64	Accellerase 1500	[17]
5	*Parthenium *	Biological	455.81	Accellerase 1500	[18]
6	*Parthenium *	Ammonia	152.28	*Aspergillus candidus* crude enzyme	[19]
7	*Parthenium *	Alkali	574.00	Accellerase 1500	Present work
