# Supplementary material for: Optimization of Enzymatic Saccharification of Alkali Pretreated Parthenium sp. Using Response Surface Methodology
Source: Enzyme Res. 2014 May 12;2014:764898. doi: 10.1155/2014/764898 (PMC4036719; doi:10.1155/2014/764898)

Supplementary Figure 1: HPLC chromatogram showing sugar release (glucose, xylose and arabinose) from *Parthenium* after saccharification under optimized condition.

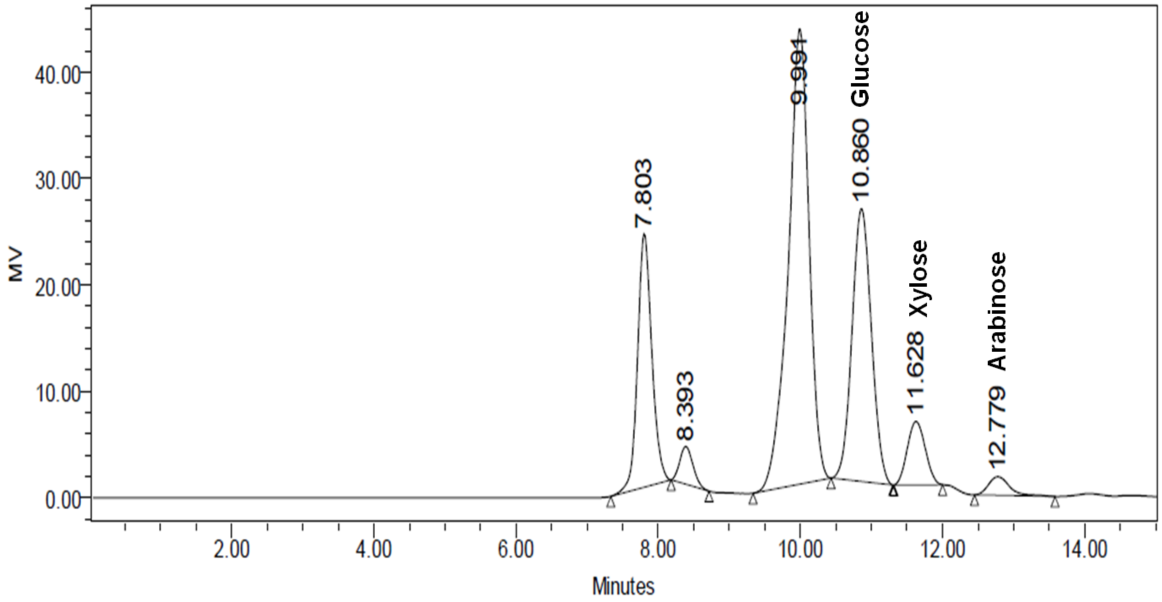

Supplement: Supplementary file 1 — The optimized condition predicted by RSM was validated to evaluate saccharification yield (%) of pretreated Parthenium sp. The HPLC analysis of saccharified hydrolysate under optimized condition showing the presence of three major peaks corresponding to glucose, xylose and arabinose. The chromatogram also represents the major contribution of glucose in the hydrolysate followed by xylose and arabinose. The quantitative analysis showing the 85.80% of saccharification yield which is in good agreement with the predicted value (80.08%). [file 764898.f1.pdf]
